# Reactivity Ratios of Biobased Dialkyl Itaconate Radical Polymerizations Derived from In-Line NMR Spectroscopy and Size-Exclusion Chromatography

**DOI:** 10.1021/acspolymersau.4c00071

**Published:** 2024-11-26

**Authors:** Marco Drache, Brunette Audree Tameno Kouanwo, Jan Christoph Namyslo, Sacha Pérocheau Arnaud, Tobias Robert, Sabine Beuermann

**Affiliations:** † Institute of Technical Chemistry, Clausthal University of Technology, Arnold-Sommerfeld-Strasse 4, 38678 Clausthal-Zellerfeld, Germany; ‡ Institute of Organic Chemistry, Clausthal University of Technology, Leibnizstrasse 6, 38678 Clausthal-Zellerfeld, Germany; § Fraunhofer Institute for Wood Research—Wilhelm-Klauditz-Institut WKI, Riedenkamp 3, 38108 Braunschweig, Germany

**Keywords:** itaconate copolymerization, depropagation, copolymerization parameter, in-line NMR, biobased monomer

## Abstract

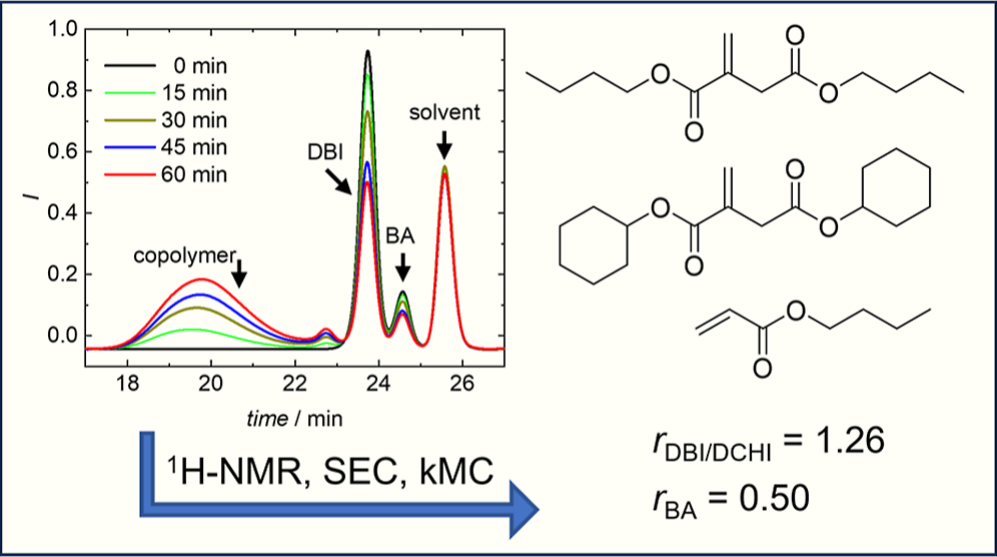

Itaconates available from renewable resources constitute a group of monomers that are used in several types of polymerizations. Their use in free-radical polymerizations (FRPs) is still limited due to the low propagation rate coefficients resulting in low polymerization rates and the occurrence of depropagation which is responsible for limited monomer conversion. Since FRP is considered very robust with few requirements concerning monomer purity, it is still interesting to investigate how itaconate FRP may become feasible. For this reason, copolymerizations of itaconates with other monomers well-suited for FRP are considered. In particular, copolymerization with acrylates appears to be interesting because the propagation rate of these monomers is high and depropagation is not operative at common polymerization temperatures. Copolymerizations of dibutyl and dicyclohexyl itaconate with butyl acrylate were performed to determine the copolymerization reactivity ratios required for tailoring copolymer composition. To limit the number of experiments, copolymerizations were carried out until high conversion and consumption of the individual monomers was obtained from ^1^H NMR spectroscopy and quantitative size-exclusion chromatography.

## Introduction

For the replacement of petrochemical-based chemicals by biobased chemicals, two strategies are considered:^1^ the so-called drop-in method, where only the origin of a component is varied. Due to the identical structure of the molecules, the same industrial processes used already in the past are feasible. Second, new components, e.g., monomers or solvents, are used. In the latter case, the chemical processes must be adapted to account for the different chemical reactivities of the substances. In the case of polymerizations, the second approach often considers biobased monomers, which are suited for polycondensations or polyadditions.^2,3^ With respect to robust polymerization processes, it is highly desirable to use biobased monomers suited for radical polymerizations, which are not sensitive to the presence of water or impurities. Moreover, radical polymerizations are attractive because they can be performed in emulsions, which is associated with avoiding the use of organic solvents, achieving excellent heat control, and good mixing due to a moderate increase in viscosity.

**Scheme 1:**
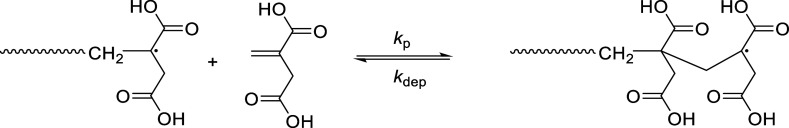
Equilibrium of Propagation and Depropagation Reactions in Itaconate Radical Polymerizations

Recently, the group of itaconic acid-based esters is gaining attention.^4^ Itaconic acid becomes available from biotechnological processes in industrial quantities at competitive prices.^5,6^ Moreover, it was referred to as one of the ten most important platform chemicals.^7^ Upon esterification of the acid, itaconic esters are available that are suited for radical polymerizations. Depending on the source of the alcohol used for esterification the biobased content of the itaconate monomers varies.^8^ As summarized by Sollka and Lienkamp,^4^ the rate of itaconate free-radical homopolymerizations and corresponding molar masses are very low. The reason may be seen in generally very low propagation rate coefficients (*k*_p_) mostly below 10 L·mol^–1^·s^–1^,^9−12^ which are more than 2 orders of magnitude lower than *k*_p_ of methacrylates. In addition, transfer rate coefficients to monomer and solvents are higher than for (meth)acrylate monomers.^13−15^ Further, early on, it was recognized that equilibrium of itaconate propagation and depropagation, as illustrated in Scheme 1 is important. Depropagation was reported to be significant for temperatures >60 °C,^9^ which contributes to low polymerization rate and limits the monomer conversion accessibility.

In order to increase polymerization rate and polymer molar masses, it appears highly rewarding to perform copolymerizations with other monomers, e.g., esters of acrylic and methacrylic acids. Although the production of both acids from biobased resources is not yet industrially relevant,^16^ several promising strategies were reported, and it appears to be a matter of time that they will become available.^17^ Upon esterification with biobased alcohols, renewable acrylates and methacrylates will be accessible. Previously, it was shown that copolymerizations of itaconates with styrene or methyl methacrylate are feasible.^18,19^ However, it is favorable to use acrylates, which are known to polymerize more quickly^20^ and do not undergo depropagation in contrast to itaconates and methacrylates.^20,21^ First results were reported for high-temperature semibatch polymerizations of the dibutyl itaconate (DBI)–butyl acrylate (BA) system.^1,22^

To allow for synthesis of materials with targeted copolymer composition, the knowledge of copolymerization reactivity ratios is essential. This is particularly true for copolymerization systems with one monomer that may undergo depropagation. However, so far this type of systems was rarely investigated, e.g., with styrene and butyl methacrylate as comonomers.^23^ Only recently, the determination of reactivity ratios for various systems with contributions from depropagation was addressed in detail.^24^

Mostly, reactivity ratios are derived from copolymerizations carried out up to low degrees of conversion.^25^ Alternatively, reactivity ratios are accessible from copolymerizations up to high conversion as long as the conversion is known. In a recent publication by an IUPAC project group entitled “Experimental Methods and Data Evaluation Procedures for the Determination of Radical Copolymerization Reactivity Ratios”, the recommendations include performing copolymerizations with widely varying monomer feed compositions and carrying out the reactions over an extended conversion range.^25^ For this approach, in-line monitoring of the individual conversions of both comonomers is particularly suited.^22,26,27^

In this contribution, copolymerizations of DBI or dicyclohexyl itaconate (DCHI) with BA covering itaconated monomer feed ratios from 0.1 to 0.9 are addressed. The individual conversion of each monomer as a function of time is derived from in-line ^1^H NMR spectroscopy. In addition, off-line size-exclusion chromatography (SEC) of the reaction mixture yields the individual residual comonomer content in the reaction mixture as a function of time. Monte Carlo (MC) simulations involving the composition data and accounting for the depropagation of the itaconate monomer units were applied to determine the reactivity ratios.

## Materials and Experimental Methods

### Materials

The monomer BA (99+% Acros Organics) was distilled prior to polymerization, and DCHI was synthesized as detailed elsewhere.^28^ DBI (>97.0%, TCI), 1,4-dioxane-*d*_8_ (Deutero GmbH), 1,4-dioxane (99.8%, water free, Sigma-Aldrich), 2,2′-azobis(isobutyronitrile) (AIBN, >98% Sigma-Aldrich), and tetrahydrofuran (THF, 99%, Grüssing) were used as received.

### Size-Exclusion Chromatography

SEC analyses were performed using a system consisting of a Knauer Marathon autosampler, a Waters 515 HPLC pump, a Knauer Smartline RI detector 2300, and a set of three chromatographic columns [100, 1000, and 100,000 Å SDV, polymer standards service (PSS)]. THF was used as an eluent with a flow rate of 1 mL·min^–1^. Calibration was established using seven polystyrene standards with molar masses ranging from 162 to 2.57 × 10^6^ g·mol^–1^ (PSS). Samples with a polymer concentration of 2 mg·mL^–1^ in THF were filtered with a syringe filter (0.45 μm) prior to injection of a sample volume of 100 μL.

### NMR Spectra

^1^H NMR spectra were recorded on a Bruker Avance III 600 MHz spectrometer (Bruker BioSpin GmbH & Co. KG, Ettlingen, Germany). Reference spectra were obtained at 25 and 60 °C. All spectral series monitoring polymerization reactions were conducted at 60 °C. Proton chemical shifts were reported in ppm relative to the residual solvent protons in 1,4-dioxane-*d*_8_ at 3.53 ppm. Multiplicities are described using the following abbreviations: s = singlet, d = doublet, t = triplet, q = quartet, and m = multiplet. Coupling constants are given in Hertz (Hz).

### Polymerization with In-Line ^1^H NMR Monitoring and Measurement Details

Polymerizations were performed directly in an ^1^H NMR tube with 0.5 mL of the reaction mixture. The thus-prepared tube was placed in the NMR spectrometer whose probe head had been preheated to 60 °C, and then a series of 1000 single-scan spectra was measured within 4 h with a frequency of 1 spectrum per 15 s (5 s prescan-delay). In addition, polymerizations were carried out in NMR tubes placed in a heating bath. The sample preparation was the same as for in-line ^1^H NMR measurements. After reaction times of 60, 120, and 180 min, the polymerizations were stopped by cooling to around 0 °C and a subsequent ^1^H NMR measurement at 60 °C was performed. Prior to polymerization and after the selected reaction time, three NMR spectra were measured. The composition of each reaction mixture is given in Table 1.

**Table 1:** Composition of Copolymerization Reaction Mixtures of BA and Either DBI or DCHI in Dioxane-*d*_8_ for Copolymerizations at 60 °C and ^1^H NMR Analyses^a^

sample	itaconate	*c*_i_/(mol·L^–1^)	*c*_BA_/(mol·L^–1^)	*f*_i_^0^	*c*_AIBN_/(mmol·L^–1^)
#1^a^	DBI	0.54	4.84	0.10	108
#2^b^	DBI	1.20	2.79	0.30	80
#3^b^	DCHI	1.20	2.79	0.30	80
#4^b^	DBI	2.09	2.09	0.50	84
#5^b^	DCHI	2.09	2.09	0.50	84
#6^a^	DBI	2.35	1.57	0.60	78
#7^b^	DBI	2.44	1.05	0.70	70
#8^a^	DBI	3.07	0.77	0.80	77
#9^a^	DBI	3.14	0.35	0.90	70

a (a) and (b) refer to off-line and in-line NMR analyses, respectively. *c*_i_, *c*_BA_, and *c*_AIBN_ refer to the initial concentrations of itaconate, BA, and AIBN, respectively. *f*_i_ is the initial molar fraction of itaconate in the reaction mixture.

### Copolymerization with Off-Line SEC Analyses

Copolymerizations of DBI or DCHI with BA at 80 °C were carried out in vials with 5 mL of reaction mixture placed in a heating block (Liebisch Labortechnik Labtherm Type 5138-6201), which was placed on a circular shaker (IKA Labortechnik KS501 digital, 100 rounds per minute). Prior to the reaction, the reaction solution was purged with nitrogen for several minutes to remove any oxygen present. Typically, the reaction was stopped after selected times (15, 30, 45, 60, 90, and 110 min) by adding traces of hydroquinone dissolved in methanol. Then, 0.2 mL of the sample was diluted with 3.8 mL of THF, and 100 μL of this solution was injected for SEC analyses. The list of compositions is provided in Table S1 of the Supporting Information.

### Software for Monte Carlo Simulations

The simulation of itaconate-BA copolymerization with consideration of itaconate depropagation was implemented in C++. Input data are the initial concentrations of the monomers, the two *r* values, and the rate coefficients of propagation and depropagation. Results of the MC simulation are the concentrations of the monomers on the conversion axis. Mersenne twister^29^ was used as the random number generator for the MC simulation. To determine the *r* values using the experimental data, the MC simulation was embedded in an optimization environment for a stochastic Metropolis–Hastings optimization environment implemented in Python 3.10.^30^ The simulations were executed on a compute server with two AMD Epyc 7H12 processors and Ubuntu 20.04.5 LTS as the operating system.

## Results and Discussion

### Data Evaluation with ^1^H NMR Spectroscopy

**Figure 1:**
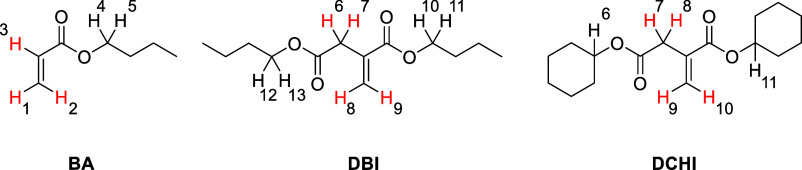
Selected protons with a focus on diagnostic resonances (in red) essential for assignment in the 600 MHz ^1^H NMR spectra of BA, DBI, and DCHI in dioxane-*d*_8_ (see detailed discussion below).

Initially, copolymerizations with varying monomer feed compositions and subsequent removal of residual monomer were carried out. However, due to the poor volatility of the itaconate monomers, the approach was highly time-consuming. Therefore, copolymerizations with an in-line ^1^H NMR measurement of monomer consumption were performed. The spectra of the itaconates DCHI and DBI show peaks assigned to olefinic protons, which are clearly separated from the olefinic protons of BA. Figure 1 provides the monomer structures.

**Figure 2:**
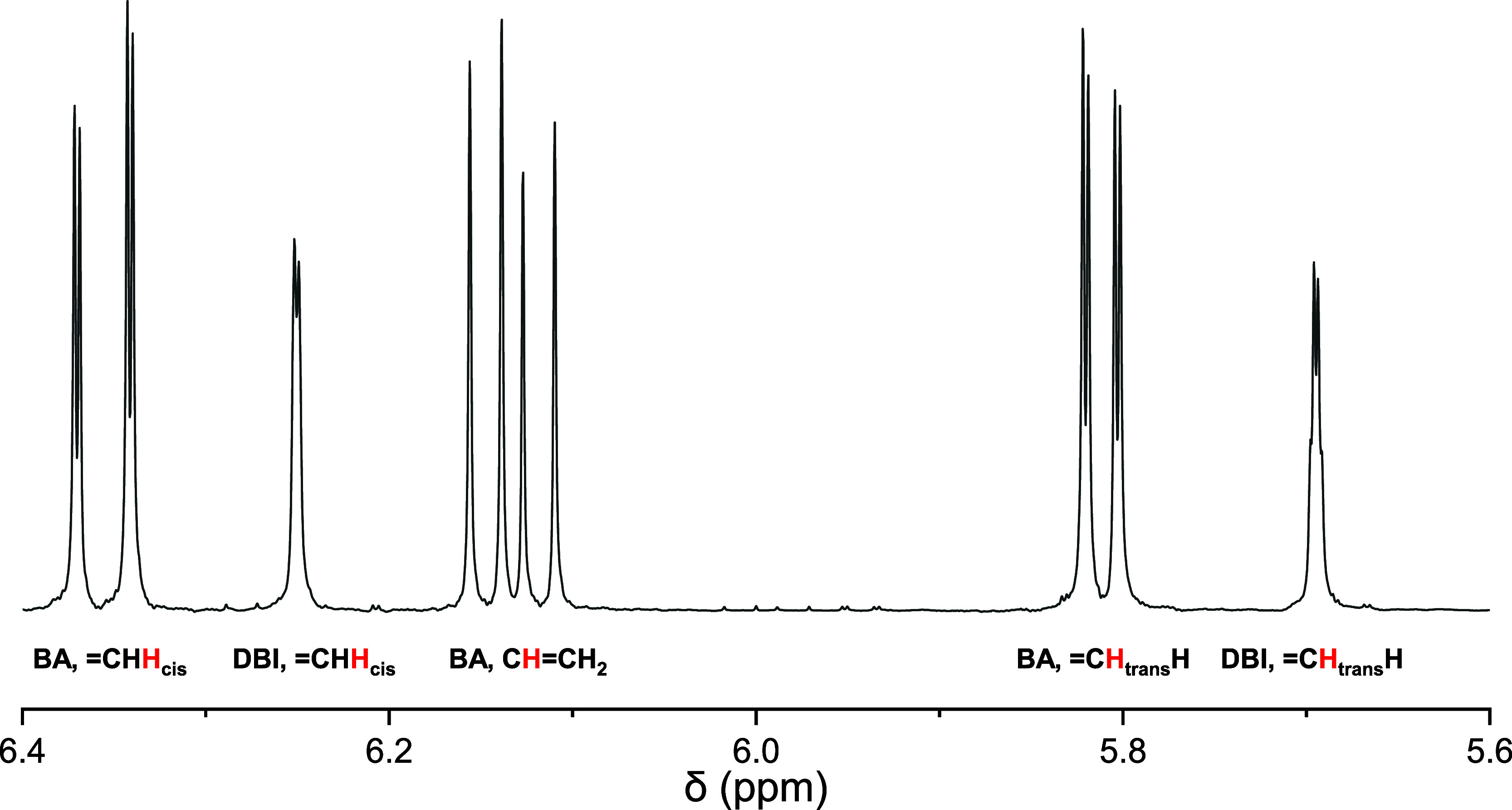
Diagnostic olefinic region of the 600 MHz NMR spectrum of a DBI-BA mixture at 25 °C (prior to copolymerization).

Even in a mixture of a symmetric itaconic acid diester (in our case DBI or DCHI) and BA in the rather uncommon deuterated solvent dioxane-*d*_8_, the assignment of the actually diagnostic protons in the olefinic region is straightforward (see Supporting Information). The most characteristic pair of signals originates from the *exo*-methylene group of such an itaconate (H-8,9 in DBI, H-9,10 in DCHI, and for comparison H-1,2 in BA, see Figures 1 and 2 as well as Figures S1 and S2 in the Supporting Information). Additionally, the more upfield located proton signal of the sp^3^ hybridized CH_2_ group adjacent to this *exo*-methylene position (H-6,7 in DBI and H-7,8 in DCHI) in the course of our NMR measurements consistently appeared as a doublet around 3.3 ppm chemical shift whose small splitting (about 1 Hz) is caused by an allylic coupling with the *trans*-proton of the *exo*-methylene. Interestingly, in previous cases, e.g., found by the groups of Robert et al.,^28^ De Vos et al.,^31^ or Palkovits et al.,^32^ this saturated methylene group within these (and other) symmetric itaconic acid derivatives was detected as a singlet.

In BA, the *cis*-olefinic proton (H-2 of BA shown in Figure 1) is found in the proton spectrum provided in Figure 2 at 6.36 ppm chemical shift as a doublet of doublets (dd) with a large *trans*-coupling of 17.4 Hz to the olefinic proton in the α-position to the carboxyl group (H-3). The doublet of doublets of the latter proton at 6.13 ppm additionally reveals a characteristic *cis*-coupling of 10.4 Hz with the second *exo*-methylene proton at 5.82 ppm (H-1) that is *trans*-positioned to the carboxyl group. Finally, the expectedly small geminal coupling of both *exo*-methylene protons is 1.8 Hz.

Appropriate diagnostic DBI signals found in the comonomer mixtures are the *exo*-methylene olefinic protons at 6.25 and 5.69 ppm. Again, the more deshielded resonance is *cis*-positioned to the carboxyl group, whereas the *trans* counterpart is found more upfield. The geminal coupling of DBI’s *exo*-methylene protons is found to be 1.3 Hz. In addition, the olefinic signal of this *trans*-proton is a doublet of triplets with the appearance of a *pseudo*-quadruplet due to partial overlap. The aforementioned allylic coupling with the saturated CH_2_ group that resonates at 3.29 ppm in the case of DBI gives a 0.8 Hz splitting. This assignment was confirmed by an appropriate set of selective saturation experiments, e.g., the olefinic resonance at 5.69 ppm (dd) collapses to a doublet (that originates from the other *exo*-methylene proton) upon saturation of the above-mentioned methylene signal at 3.29 ppm. In the case of DCHI, the diagnostic proton signals of the inherent itaconic acid core resonate at almost the same positions with almost identical spin–spin coupling constants (see NMR data and Figure S2 in the Supporting Information).

**Figure 3:**
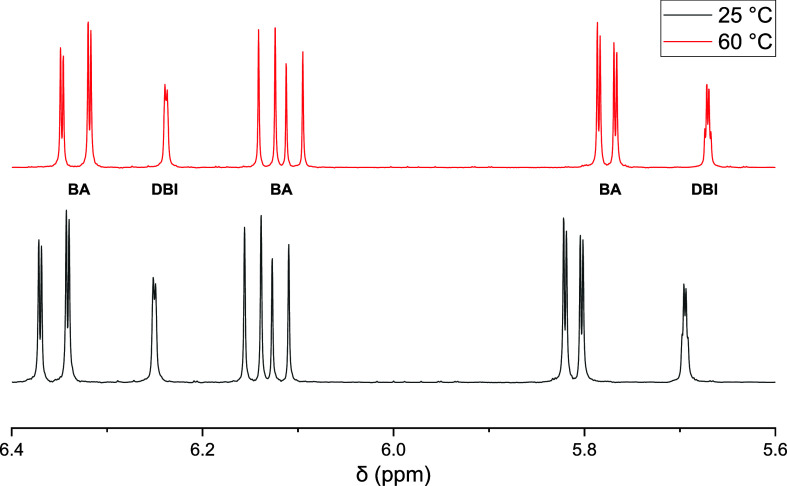
Comparative plot of the proton spectra of the DBI-BA mixture at 25 and 60 °C with only moderate temperature-dependent proton shift differences ≤0.03 ppm.

All hitherto given chemical shift values refer to measurement in dioxane-*d*_8_ at 25 °C. It should be mentioned that the said diagnostic resonances of the olefinic region between about 6.5 and 5.5 ppm lead to only small shift differences of about Δδ 0.01 to 0.03 ppm upon increase of the temperature to 60 °C (utilized for in situ copolymerizations). This is illustrated in the stacked plot of the corresponding proton spectra in Figure 3.

**Figure 4:**
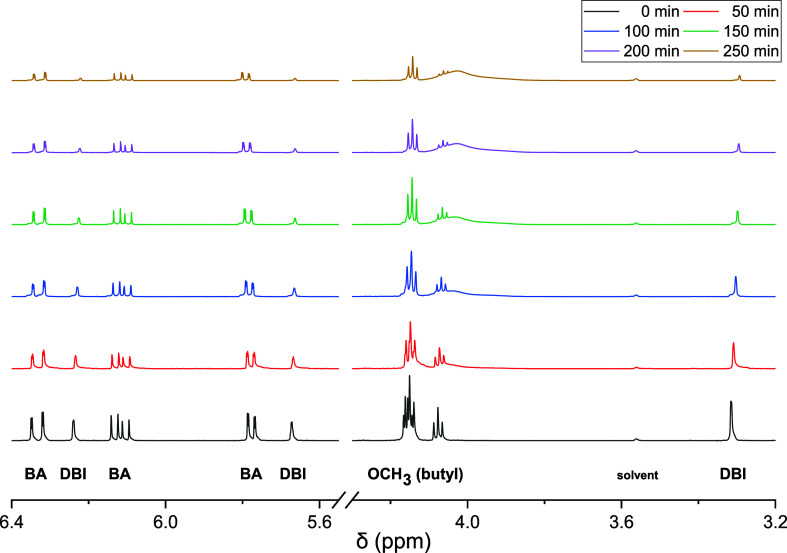
Bottom to top proton NMR monitoring of the copolymerization of DBI-BA in dioxane-*d*_8_ at 60 °C within about 4 h with *f*_DBI_^0^ = 0.30.

With these spectroscopical characteristics and assignments of the starting materials in hand, the ^1^H NMR spectra recorded during a DBI-BA copolymerization at 60 °C shown in Figure 4 were evaluated.

The decrease in the intensity of the peaks assigned to the olefinic protons is clearly seen. Similar spectra series were obtained for DBI-BA copolymerizations with initial monomer feed compositions ranging from 0.1 to 0.9. Further, two copolymerizations of DCHI and BA with *f*_DCHI_^0^ of 0.3 and 0.5 were carried out. As an example, a spectra series is provided in the Supporting Information as Figure S3.

The conversion *x* of each individual monomer is calculated according to eq 1
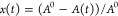
1with *A* being absolute integrals of the NMR peaks. The concentration *c*(*t*) is calculated according to eq 2
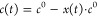
2*A*^0^ and *c*^0^ refer to the initial reaction mixture at time zero.

**Figure 5:**
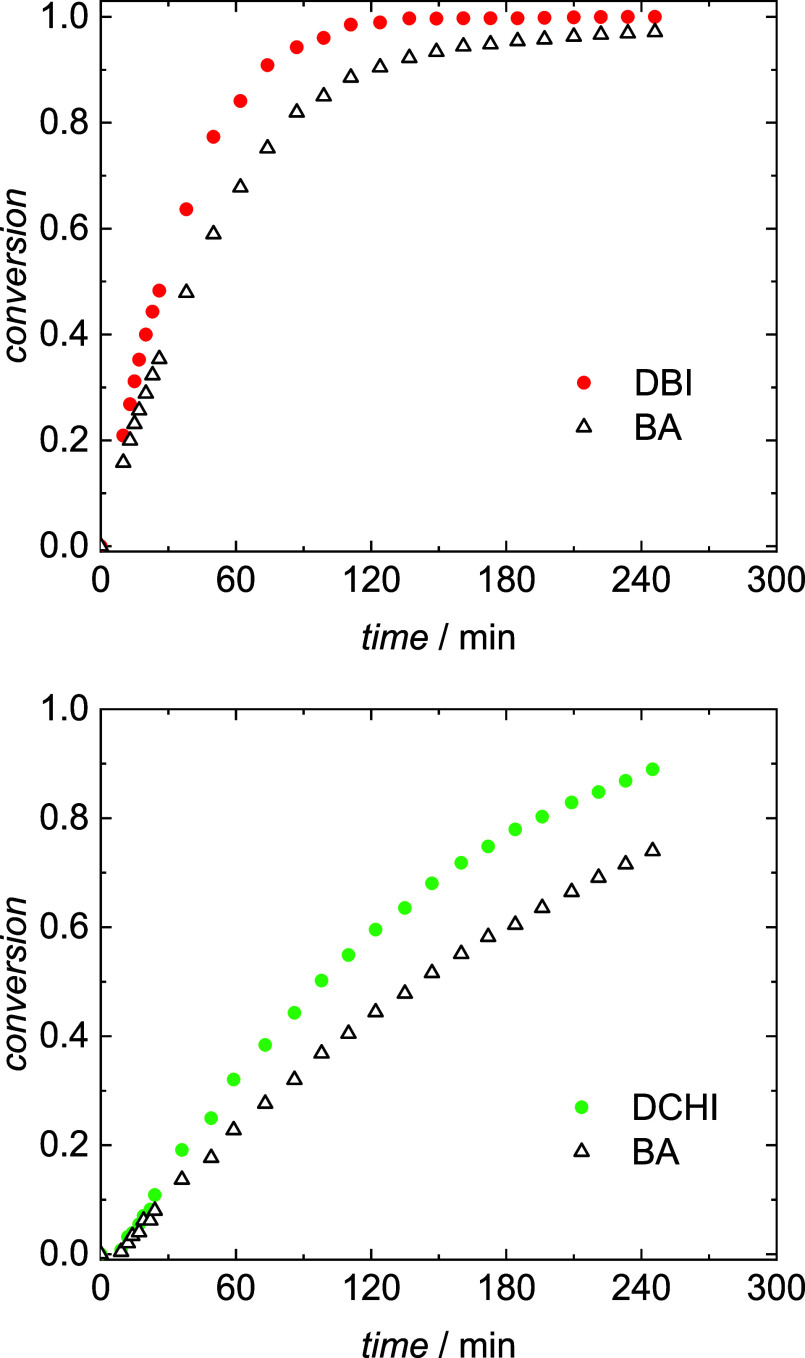
Conversion–time curves for copolymerizations of BA with DBI (top) and with DCHI (bottom) at 60 °C with *f*_DBI_^0^ = 0.30 and *f*_DCHI_^0^ = 0.30, respectively.

Figure 5 gives two examples for the variation of monomer conversion with time for DBI–BA with *f*_DBI_^0^ = 0.30 (top) and for DCHI–BA with *f*_DCHI_^0^ = 0.30 (bottom). For both systems, the conversion of the itaconate comonomer is higher than for BA throughout the polymerization, indicating preferential itaconate incorporation into the copolymer. The finding is not surprising since the itaconate monomer results in a tertiary and BA in a secondary propagating radical. Similarly, in methacrylate–acrylate systems the methacrylate monomer is preferentially built into the copolymer.^33^ With eq 2 the concentration of each monomer as a function of time is calculated.

**Figure 6:**
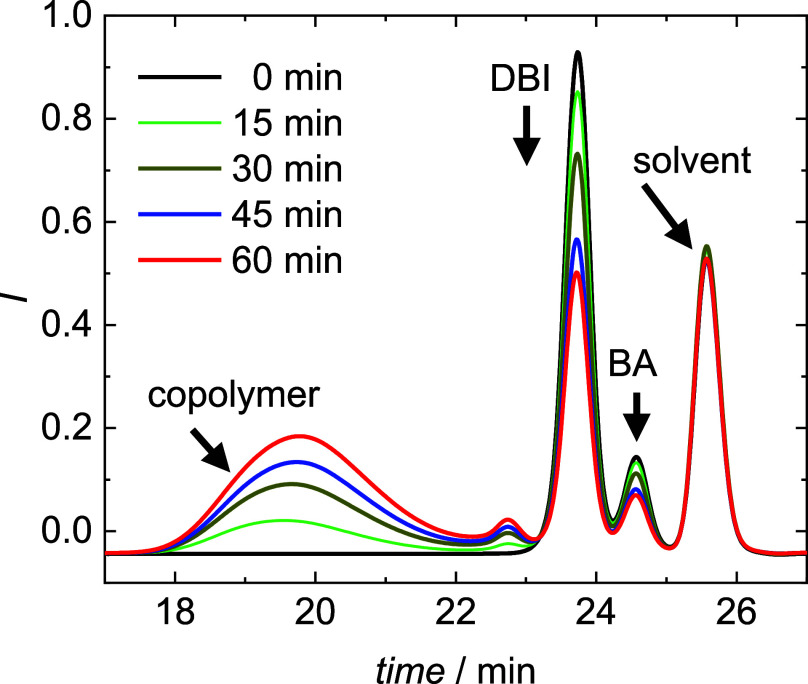
SEC elution curves for the copolymerization of DBI and BA at 80 °C with *f*_DBI_^0^ = 0.50.

In addition, copolymerizations at 80 °C were carried out in thermostated vials for various reaction times. In-line ^1^H NMR monitoring at 80 °C is not feasible; moreover, the reactions proceed too quickly. After reaching the selected polymerization time, the reaction mixture was directly injected for SEC analyses. Figure 6 shows that the peaks of the solvent, the monomers, and the copolymer are well separated in the SEC elution curves. Due to the differences in size, subsequent to the copolymer, the monomer DBI elutes first, followed by BA and finally dioxane. Since the concentration of the solvent does not change during the polymerization, the area of the dioxane peak remains constant and serves as an internal reference. As expected, the area of both monomer peaks decreases and can be used to determine the monomer conversion of each monomer. In addition, a small peak around 22.7 min is seen, which is suggested to be due to products from side reactions such as backbiting and subsequent scission, which were reported for both monomers.^1,20,34^ Additionally, bimodality of molar mass distributions was observed in styrene–methacrylate copolymerizations at high temperatures with significant contributions from methacrylate depropagation.^35^ To identify the origin of the peak occurring around 22.7 min in Figure 6, electrospray ionization mass spectrometry (ESI-MS) analyses of the low molar mass material was planned, which was already applied to products from BA homopolymerizations.^34^

The monomer peak areas were determined by means of fitting each peak with Gaussian curves using the program Origin.^36^Figure S4 of the Supporting Information shows that the peak areas change linearly with the monomer content. In analogy to the interpretation of the NMR experiments using eqs 1 and 2, the monomer conversion can be calculated from the peak area before the reaction and at a defined reaction time.

### Modeling Strategy

**Scheme 2:**
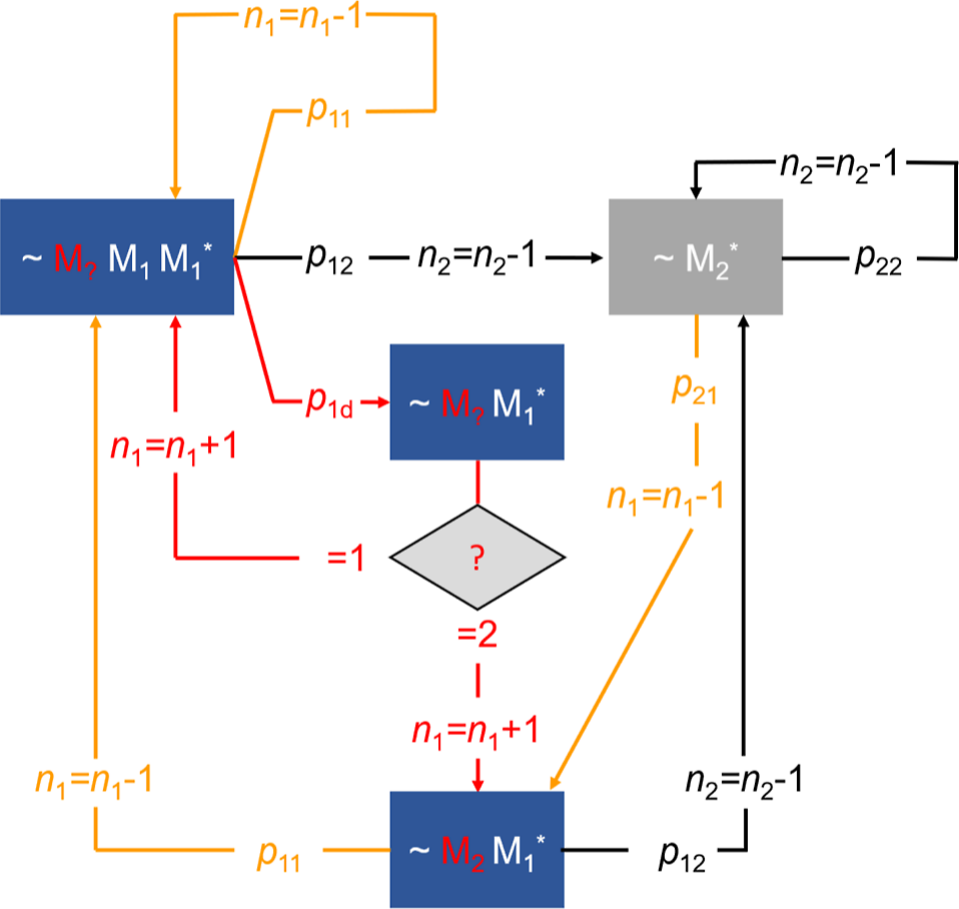
MC Simulation of Chain Propagation in Copolymerizations of DBI (1) and BA (2) Accounting for Depropagation of Monomer 1 The asterisk marks the terminal monomer unit of the radical. The other acronyms are explained in the main text. The possible reaction pathways (addition of DBI, BA, and depropagation) have been color-coded: orange color refers to propagation of DBI units, black color to propagation of BA units, and red color to depropagation of DBI units.

In order to derive the reactivity ratios, a MC modeling approach was applied, which considers the competing reactions at the macroradical chain end. It should be noted that the MC simulations do not account for the full kinetic scheme, and thus, e.g., reaction rates are not accessible. A comparable method was already applied to evaluate transfer processes and the temperature dependence of defect structures occurring in vinylidene fluoride iodine transfer polymerizations.^37,38^ The approach applied to determine the reactivity ratios considering itaconate depropagation is illustrated in Scheme 2. The kinetic coefficients used are listed in Table S2, and the underlying mechanism is provided in Scheme S2 of the Supporting Information. First, the number of molecules *n*_1_ and *n*_2_ is initialized according to the comonomer feed composition, with index 1 referring to DBI and index 2 referring to BA. A total of 10^8^ monomer molecules is used. The simulation shown in Scheme 2 starts with BA at the chain end of a growing macroradical (∼M_2_*). The macroradicals may undergo homopolymerization with BA (M2) or copolymerization with DBI (M1). The corresponding reaction probabilities are calculated using eqs 3 and 4.
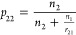
3
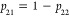
4

The selection of the reaction path is performed on the basis of reaction probabilities and using a random number, *rn* (0 ≤ *rn* ≤ 1). In the case of homopropagation, the chain end remains unchanged, and one BA molecule is consumed, which is expressed by the assignment (*n*_2_ = *n*_2_ – 1). Thus, the comonomer feed composition is changed. If a cross-propagation reaction with DBI is selected, the chain end of the macroradical changes to DBI in the terminal position and BA in the penultimate position (∼M_2_ M_1_*) and the comonomer feed is altered with the consumption of a DBI monomer molecule expressed by the assignment (*n*_1_ = *n*_1_ – 1).

#### Chain End ∼ M_2_ M_1_*

Since a BA unit is situated at the penultimate position of the macroradical, depropagation of DBI is excluded, and only homopropagation with DBI or cross propagation with BA is possible. After calculating the reaction probabilities *p*_11_ and *p*_12_ (eqs 5 and 6), the reaction path is also selected using a random number.
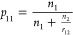
5
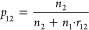
6

Cross propagation restores the terminal BA unit in the macroradical (∼M_2_*), and a BA monomer molecule is consumed (*n*_2_ = *n*_2_ – 1). Homopropagation with DBI results in the consumption of DBI (*n*_1_ = *n*_1_ – 1) and the sequence ∼M_2_ M_1_ M_1_* at the chain end. The length of the DBI sequence at the end of the macroradical is logged in the simulation.

#### Chain End ∼ M_?_ M_1_ M_1_*

Since two or more DBI monomers are located at the end of the chain, DBI depropagation can occur in competition with addition to a DBI or a BA molecule. The monomer M_?_ is either DBI (? = 1) or BA (? = 2). Homopropagation with DBI extends, and depropagation of DBI decreases the DBI sequence at the end of the macroradical. The reaction probabilities *p*_11_ and *p*_12_ for homo- and cross propagation are calculated using eqs 5 and 6. In addition, the ratio of DBI propagation to depropagation can be calculated by using eq 7.
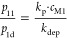
7
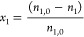
8
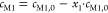
9

The current monomer conversion *x*_1_ of DBI and *x*_2_ of BA is calculated using the initial and current counters *n*_1_ and *n*_2_ according to eq 8. The concentration of DBI, *c*_M1_, is calculated from the initial DBI concentration, *c*_M1,0_ and *x*_1_ with eq 9. After the current reaction probabilities *p*_11_, *p*_12_, and *p*_dep_, a random number is used to select the reaction path. The homopropagation consumes one DBI molecule (*n*_1_ = *n*_1_ – 1) and extends the DBI sequence at the chain end. The cross-propagation reaction pathway consumes one BA molecule (*n*_2_ = *n*_2_ – 1), and BA is localized at the chain end (∼M_2_*). The depropagation shortens the DBI sequence at the chain end, and the number of DBI molecules increases (*n*_1_ = *n*_1_ + 1). Now, the penultimate monomer unit at the end of the macroradical (M_?_) is considered. In the case of DBI (? = 1), the chain end remains unchanged (∼M_?_ M_1_ M_1_*); in the case of BA (? = 2), the depropagation pathway is not applicable and the next reaction step is calculated with a BA unit at the penultimate position (∼M_2_ M_1_*).

The monomer conversion is calculated from the number of molecules *n*_1_ and *n*_2_ after each reaction step, and the actual monomer concentrations are output after changes in conversion of 0.01. In addition, the number of propagation and depropagation steps carried out are logged. The simulation ends at a monomer conversion of 0.99 or when an equilibrium of propagation and depropagation is reached. Typically, a simulation takes 3–4 s of computing time. The relative concentrations *c*_1_ and *c*_2_ can be directly compared with the experimental data from the NMR experiments. Regardless of the initial composition of the reaction mixture *f*_1_^0^, only the itaconate reaction rate coefficients *k*_p_ and *k*_dep_ known from the literature^11^ and the reactivity ratios *r*_1_ and *r*_2_ are required for the description of the concentration data. The unknown *r*_1_ and *r*_2_ parameters are obtained via MC simulations applying a Metropolis Hastings method,^39^ which was applied previously.^30,38^

**Figure 7:**
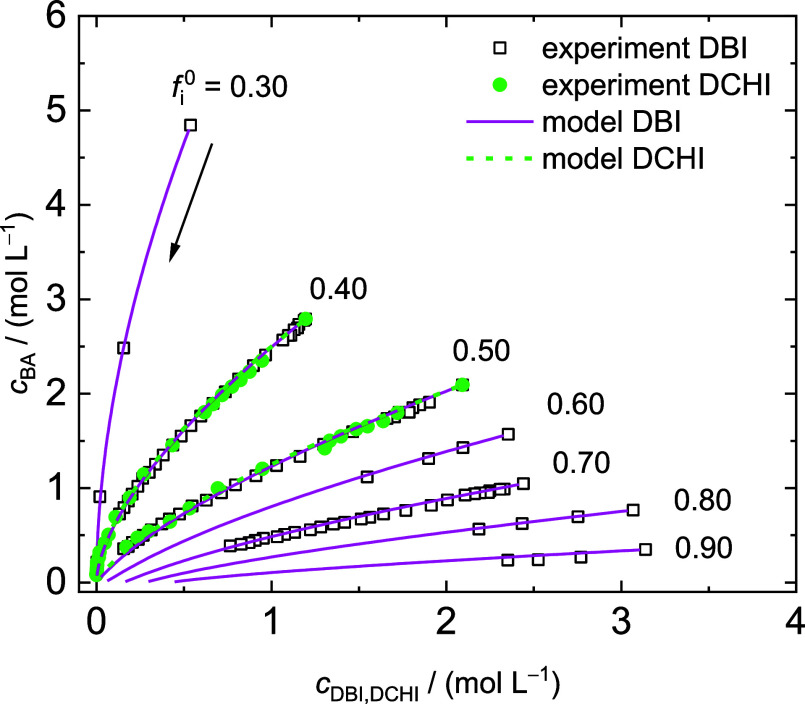
Variation of BA concentration as a function of itaconate concentration for copolymerizations of DBI and DCHI at 60 °C derived from NMR measurements. Initial itaconate feed ratios, *f*_i_^0^, are indicated. The markers refer to experimental data and the lines to results from MC simulations with *r*_DBI,DCHI_ = 1.26 and *r*_BA_ = 0.50. Polymerization proceeds toward low concentrations, as illustrated by the arrow for *f*_DBI_^0^ = 0.30. The experimental data is given in the Supporting Information.

Figure 7 provides the relative concentration profiles for copolymerization at 60 °C for both monomer systems, DBI–BA and DCHI–BA, with the BA concentration plotted as a function of itaconate concentration. It is seen that the relative concentration profiles for both monomer systems cannot be distinguished for the initial itaconate feed compositions *f*_1_^0^ = 0.40 and *f*_1_^0^ = 0.50. Therefore, common *r* values for both comonomer systems were determined by fitting the copolymerization model illustrated in Scheme 2 to the experimental data shown in Figure 7. A Metropolis–Hastings method was applied to the fit. The resulting *r* values for the itaconates and BA are *r*_DBI,DCHI_ = 1.26 and *r*_BA_ = 0.50, respectively.

**Figure 8:**
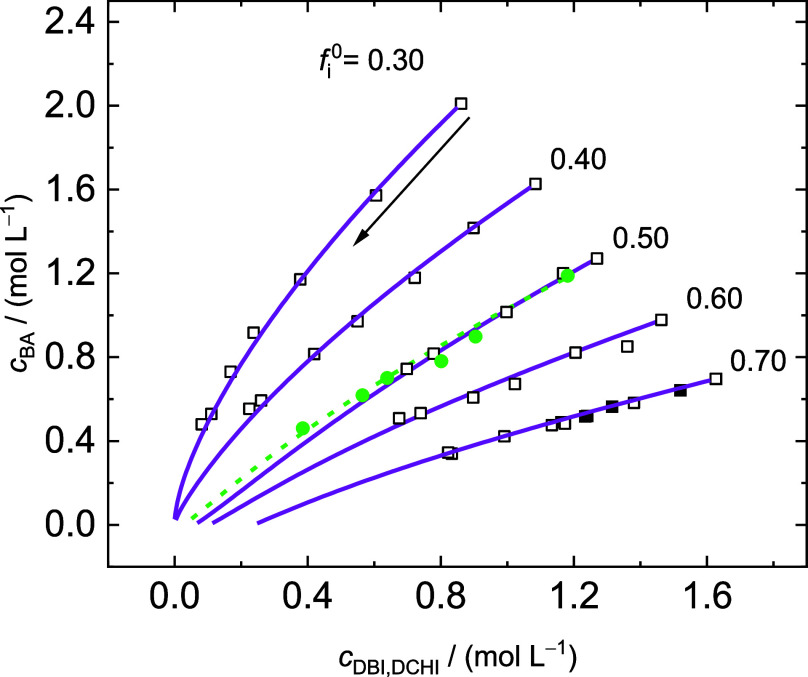
Variation of BA concentration with itaconate concentration during copolymerizations of BA with DBI (black squares) or DCHI (green circles) at 80 °C calculated from residual monomer concentrations derived from off-line SEC analyses. The symbols refer to experimental data, and the lines represent the results from the MC simulations using the reactivity ratios *r*_DBI,DCHI_ = 1.26 and *r*_BA_ = 0.50. Polymerization proceeds toward low concentrations, as illustrated by the arrow. The experimental data is given in the Supporting Information.

Figure 8 presents the concentration data for DBI/BA copolymerizations at 80 °C. The markers refer to experimental data derived from off-line SEC analyses. The lines given in Figure 8 represent the simulation results, which were obtained with the *r* values derived for 60 °C, and the temperature dependent DBI kinetic coefficients for homopropagation and depropagation.^10,11^ Very good agreement between the simulated and experimental data is found. It was assumed that the *r* values are not significantly varied by the temperature because of the rather narrow temperature interval of the experiments. In addition, data for DCHI/BA copolymerization at 80 °C with *f*_DCHI_ = 0.50 is contained. Again, the experimental data is very well represented by the simulations given by the dashed line. The results show that the *r* values determined at 60 °C can also be applied to polymerizations at 80 °C and, consequently, more pronounced depropagation.

**Figure 9:**
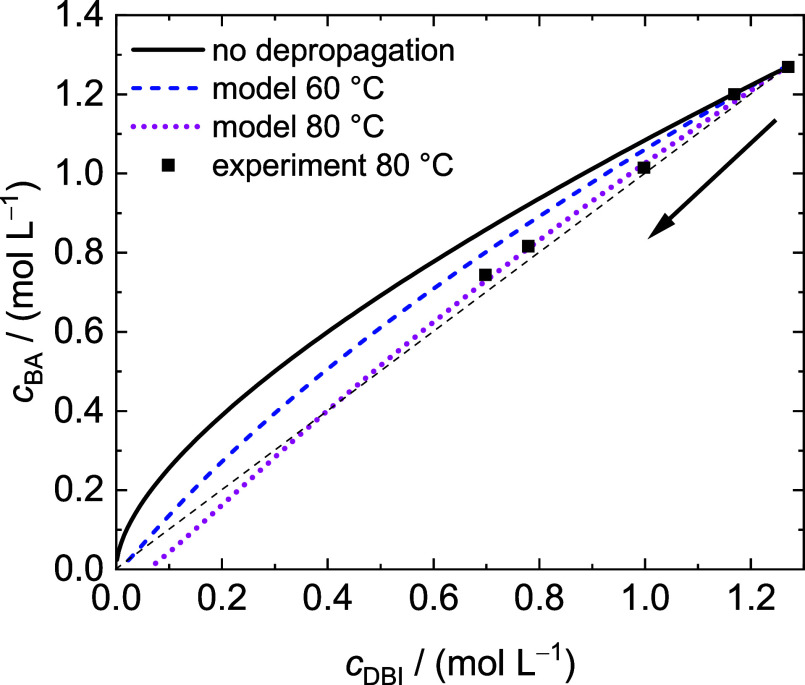
Variation of BA concentration as a function of DBI concentration during copolymerizations with *f*_DBI_^0^ = 0.50. The squares refer to experimental data obtained at 80 °C, and the lines represent the results from the MC simulations at 60 and 80 °C using the reactivity ratios *r*_DBI,DCHI_ = 1.26 and *r*_BA_ = 0.50 and temperature-dependent *k*_p_ and *k*_dep_ values. Polymerization proceeds toward low concentrations, as illustrated by the arrow. The diagonal is indicated by the black dashed line.

DBI–BA data derived from simulations for temperatures ranging from 60 to 80 °C are considered in Figure 9. The data demonstrate the influence of DBI depropagation on the DBI/BA copolymerization. At 60 °C, DBI is preferentially incorporated into the copolymer, which is indicated by the modeled data for *c*_BA_ being above the diagonal (dashed black line). With increasing temperature, at 80 °C the incorporation of BA becomes favored, regardless of unchanged *r* values. Once all BA is consumed, polymerization stops although a DBI concentration of 0.07 mol/L remains at 80 °C. This finding is due to depropagation.

**Figure 10:**
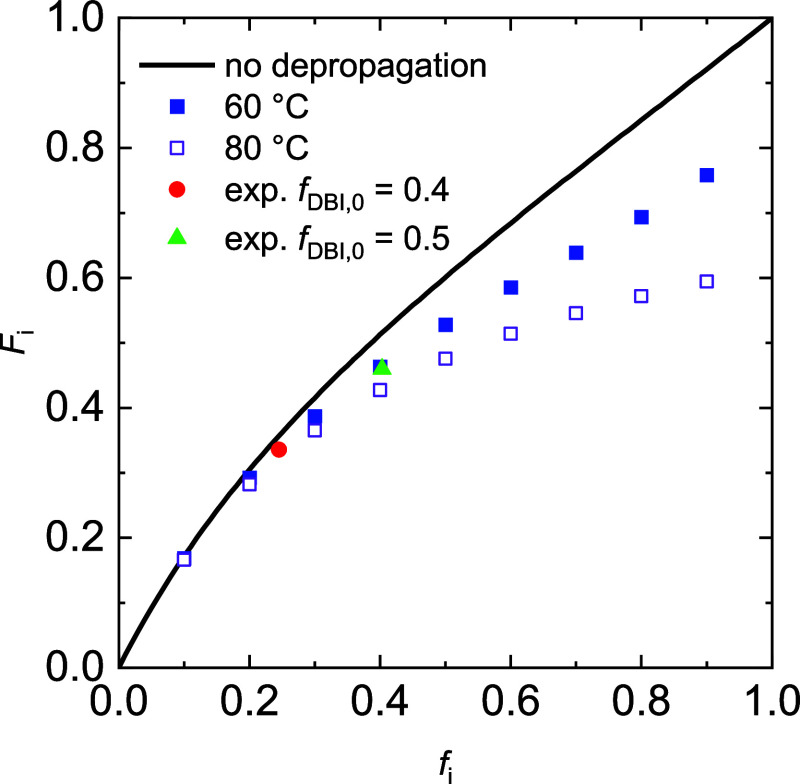
Variation of molar itaconate content in the copolymer, *F*_i_, with the itaconate content in the monomer feed, *f*_i_. The full line refers to copolymerization without depropagation and the markers to cases with depropagation. The data points referring to systems with depropagation were calculated with eq 10 and are given by the squares; the circle and triangle represent experimental data. Further details are given in the main text.

The representations in Figures 7–9 were chosen to account for the influence of the itaconate concentration on the depropagation. To illustrate how the data may be related to a commonly used copolymerization diagram, Figure 10 provides the itaconate content in the copolymer, *F*_i_, as a function of the itaconate content in the monomer mixture, *f*_i_. Using the reactivity ratios of 1.26 and 0.50 determined for itaconate and BA, respectively, and eq 10(25) the full line was obtained.
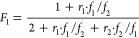
10Subscripts 1 and 2 refer to the itaconate monomer and BA, respectively.

This full line in Figure 10 represents systems without any depropagation. Since concentration of the monomer undergoing depropagation has a large impact on the extent of depropagation,^21^ the data points referring to systems with depropagation were obtained as follows: the itaconate concentration was fixed at 0.5 mol·L^–1^ and the corresponding BA concentration was calculated according to the selected monomer feed concentrations. Then, MC simulations accounting for the temperature dependence of the propagation and depropagation rate coefficients were carried out. The code is provided in the Supporting Information. The use of eq 10 is not feasible since the impact of the itaconate concentration on depropagation cannot be accounted for. Figure 10 shows only a small difference between all data for low *f*_i_. A significant difference between the data obtained with consideration of depropagation compared with the case without any depropagation is found for higher itaconate contents in the monomer mixture. As expected, the difference is larger the higher the temperature. Figure 10 illustrates that it is important to limit the temperature in copolymerizations aiming for high contents of itaconates from biobased resources in the copolymer.

For comparison of the calculated data in Figure 10 with experimentally derived data, two data points were added. The experiments selected were carried out with *f*_i_^0^ = 0.4 and 0.5. These initial itaconate monomer feed compositions are associated with itaconate concentrations of 0.54–0.48 and 0.55–0.47 mol·L^–1^, respectively, during the course of the copolymerization. These values are rather close to 0.5 mol·L^–1^ used for the calculation of the data points in Figure 10. Very good agreement of all data is seen.

## Conclusions

Itaconic acid and its esters make up an interesting class of monomers available from renewable resources. However, radical polymerizations of itaconates are characterized by very low propagation rate coefficients and depropagation becoming influential at temperatures as low as 60 °C. Despite this unfavorable aspect, it appears attractive to use these monomers in very robust radical polymerization processes. Unlike those important for other polymerization methods, extensive purification and drying of the monomer is not required. To overcome the generally slow radical polymerization rates, copolymerization with fast propagating acrylates is attractive. To tailor the copolymer composition, the knowledge of the reactivity ratios is highly valuable.

Here, copolymerizations of the DBI/BA and DCHI/BA monomer systems were investigated. The copolymerizations carried out until high monomer conversion were analyzed with (in-line) ^1^H NMR spectroscopy or quantitative SEC analyses of the residual reaction mixtures, thus limiting the number of experiments required. In all cases, preferential incorporation of the itaconate monomers was observed. Moreover, the reaction rate is significantly enhanced due to the presence of the acrylate monomer. Applying MC simulations using propagation and depropagation rate coefficients for the corresponding homopolymerization systems from the literature, it is found that both comonomer systems are represented by a common set of reactivity ratios: *r*_DBI/DCHI_ = 1.26 and *r*_BA_ = 0.50. Considering the large difference between itaconate and acrylate homopolymerization *k*_p_ values of more than 3 orders of magnitude and the rather small variation in *k*_p_ caused by the type of ester group within the itaconate and the acrylate monomer group, the result suggests that these *r* values may be used to estimate the copolymer composition for other monomer systems with alkyl ester groups as well.

## Supporting Information Available

Copolymerization reaction mixtures at 80 °C, ^1^H NMR spectroscopy details, kinetic data and reaction scheme for MC simulations, program code files, and tables with experimental data (ZIP)

## References

[ref1] Pirman T.; Sanders C. A.; Jasiukaitytė Grojzdek E.; Lazic V.; Ocepek M.; Cunningham M. F.; Likozar B.; Hutchinson R. A. Free-Radical Homopolymerization Kinetics of Biobased Dibutyl Itaconate. ACS Appl. Polym. Mater. 2023, 5, 9213–9224. 10.1021/acsapm.3c01708.

[ref2] Fernandes C. D.; Oechsler B. F.; Sayer C.; de Oliveira D.; de Araujo P. H. H. Recent advances and challenges on enzymatic synthesis of biobased polyesters via polycondensation. Eur. Polym. J. 2022, 169, 111132 10.1016/j.eurpolymj.2022.111132.

[ref3] Winkler M.; Lacerda T. M.; Mack F.; Meier M. A. R. Renewable Polymers from Itaconic Acid by Polycondensation andRing-Opening-Metathesis Polymerization. Macromolecules 2015, 48, 1398–1403. 10.1021/acs.macromol.5b00052.

[ref4] Sollka L.; Lienkamp K. Progress in the Free and Controlled Radical Homo- and Co-Polymerization of Itaconic Acid Derivatives: Toward Functional Polymers with Controlled Molar Mass Distribution and Architecture. Macromol. Rapid Commun. 2021, 42, 2000546 10.1002/marc.202000546. 33270308

[ref5] Kuenz A.; Krull S. Biotechnological production of itaconic acid – things you have to know. Appl. Microbiol. Biotechnol. 2018, 102, 3901–3914. 10.1007/s00253-018-8895-7. 29536145

[ref6] Krull S.; Lünsmann M.; Prüße U.; Kuenz A. Ustilago Rabenhorstiana - An Alternative Natural Itaconic Acid Producer. Fermentation 2020, 6, 4 10.3390/fermentation6010004.

[ref7] Dai J.; Ma S.; Wu Y.; Han L.; Zhang L.; Zhu J.; Liu X. Polyesters derived from itaconic acid for the properties and bio-based content enhancement of soybean oil-based thermosets. Green Chem. 2015, 17, 2383–2392. 10.1039/C4GC02057J.

[ref8] Allasia M.; Aguirre M.; Gugliotta L. M.; Minari R. J.; Leiza J. R. High biobased content waterborne latexes stabilized with casein. Prog. Org. Coat. 2022, 168, 106870 10.1016/j.porgcoat.2022.106870.

[ref9] Sato T.; Inui S.; Tanaka H.; Ota T.; Kamachi M.; Tanaka K. Kinetic and ESR studies on the radical polymerization of Di-*n*-butyl itaconate in benzene. J. Polym. Sci., Polym. Chem. Ed. 1987, 25, 637–652. 10.1002/pola.1987.080250216.

[ref10] Kattner H.; Buback M. Propagation and Chain-Length Dependent Termination Rate Coefficients Deduced from a Single SP-PLP-EPR experiment. Macromolecules 2016, 49, 3716–3722. 10.1021/acs.macromol.6b00483.

[ref11] Szablan Z.; Stenzel M. H.; Davis T. P.; Barner L.; Barner-Kowollik C. Depropagation Kinetics of Sterically Demanding Monomers: A Pulsed Laser Size Exclusion Chromatography Study. Macromolecules 2005, 38, 5944–5954. 10.1021/ma050444l.

[ref12] Meyer E.; Weege T.; Vana P. Free-Radical Propagation Rate Coefficients of Diethyl Itaconate and Di-n-Propyl Itaconate Obtained via PLP–SEC. Polymers 2023, 15, 1345 10.3390/polym15061345. 36987126 PMC10056010

[ref13] Katsikas L.; Milovanović M.; Popović I. G. Hindered, 1,1-disubstituted monomers. Chain transfer to benzene in the radical polymerisation of di-n-butyl itaconate. Eur. Polym. J. 2008, 44, 3028–3031. 10.1016/j.eurpolymj.2008.06.039.

[ref14] Tomić S. L.; Filipović J. M.; Velicković J. S.; Katsikas L.; Popović I. G. The polymerisation kinetics of lower dialkyl itaconates. Macromol. Chem. Phys. 1999, 200, 2421–2427. 10.1002/(SICI)1521-3935(19991001)200:10<2421::AID-MACP2421>3.0.CO;2-E.

[ref15] Szablan Z.; Toy A. A.; Terrenoire A.; Davis T. P.; Stenzel M. H.; Müller A. H. E.; Barner-Kowollik C. Living free-radical polymerization of sterically hindered monomers: Improving the understanding of 1,1-disubstituted monomer systems. J. Polym. Sci., Part A: Polym. Chem. 2006, 44, 3692–3710. 10.1002/pola.21470.

[ref16] Hayes G.; Laurel M.; MacKinnon D.; Zhao T.; Houck H. A.; Becer C. R. Polymers without Petrochemicals: Sustainable Routes to Conventional Monomers. Chem. Rev. 2023, 123, 2609–2734. 10.1021/acs.chemrev.2c00354. 36227737 PMC9999446

[ref17] Fouilloux H.; Thomas C. M. Production and Polymerization of Biobased Acrylates and Analogs. Macromol. Rapid Commun. 2021, 42, 2000530 10.1002/marc.202000530. 33433958

[ref18] Madruga E. L.; Fernandez-Garcia M. Free-radical homopolymerization and copolymerization of di-*n*-butyl itaconate. Polymer 1994, 35, 4437–4442. 10.1016/0032-3861(94)90104-X.

[ref19] Fernandez-Garcia M.; Madruga E. L.; Cuervo-Rodriguez R. A kinetic study on the radical copolymerization of dimethyl itaconate and methyl methacrylate in benzene. Polymer 1996, 37, 263–268. 10.1016/0032-3861(96)81097-4.

[ref20] Ballard N.; Asua J. M. Radical polymerization of acrylic monomers: An overview. Prog. Polym. Sci. 2018, 79, 40–60. 10.1016/j.progpolymsci.2017.11.002.

[ref21] Hutchinson R. A.; Paquet D. A. Jr.; Beuermann S.; McMinn J. H. Investigation of Methacrylate Free-Radical Depropagation Kinetics by Pulsed-Laser Polymerization. Ind. Eng. Chem. Res. 1998, 37, 3567–3574. 10.1021/ie980167p.

[ref22] Pirman T.; Sanders C. A.; Ocepek M.; Cunningham M. F.; Likozar B.; Hutchinson R. A. Free radical copolymerization kinetics of bio-based dibutyl itaconate and n-butyl acrylate. Chem. Eng. J. 2024, 499, 156127 10.1016/j.cej.2024.156127.

[ref23] Li D.; Grady M. C.; Hutchinson R. A. High-Temperature Semibatch Free Radical Copolymerization of Butyl Methacrylate and Butyl Acrylate. Ind. Eng. Chem. Res. 2005, 44, 2506–2517. 10.1021/ie049651k.

[ref24] Lundberg D. J.; Kilgallon L. J.; Cooper J. C.; Starvaggi F.; Xia Y.; Johnson J. A. Accurate Determination of Reactivity Ratios for Copolymerization Reactions with Reversible Propagation Mechanisms. Macromolecules 2024, 57, 6727–6740. 10.1021/acs.macromol.4c00835.

[ref25] Autzen A. A. A.; Beuermann S.; Drache M.; Fellows C. M.; Harrisson S.; van Herk A. M.; Hutchinson R. A.; Kajiwara A.; Keddie D. J.; Klumperman B.; Russell G. T. IUPAC Recommended Experimental Methods and Data Evaluation Procedures for the Determination of Radical Copolymerization Reactivity Ratios from Composition Data. Polym. Chem. 2024, 15, 1851–1861. 10.1039/D4PY00270A.

[ref26] Möller E.; Schreiber U.; Beuermann S. In line spectroscopic investigation of fluorinated copolymer synthesis in supercritical carbon dioxide. Macromol. Symp. 2010, 289, 52–63. 10.1002/masy.200900007.

[ref27] Agboluaje M.; Kaur G.; Dušička E.; Urbanová A.; Pishnamazi M.; Horváth B.; Janata M.; Raus V.; Lacík I.; Hutchinson R. A. A systematic study of tert-butylacrylamide-methyl acrylate-acrylic acid radical solution terpolymerization. Can. J. Chem. Eng. 2023, 101, 5300–5531. 10.1002/cjce.24947.

[ref28] Pérocheau Arnaud S.; Malitowski N. M.; Meza Casamayor K.; Robert T. Itaconic acid-based reactive diluents for renewable and acrylate-free UV-curing additive manufacturing materials. ACS Sustainable Chem. Eng. 2021, 9, 17142–17151. 10.1021/acssuschemeng.1c06713.

[ref29] Matsumoto M.; Nishimura T. Mersenne twister: A 623-dimensionally equidistributed uniform pseudorandom number generator. ACM Trans. Model. Comput. Simul. 1998, 8, 3–30. 10.1145/272991.272995.

[ref30] Feuerpfeil A.; Drache M.; Jantke L.-A.; Melchin T.; Rodríguez-Fernández J.; Beuermann S. Modeling Semi-Batch Vinyl Acetate Polymerization Processes. Ind. Eng. Chem. Res. 2021, 60, 18256–18267. 10.1021/acs.iecr.1c03114.

[ref31] Verduyckt J.; Geers A.; Claes B.; Eyley S.; Van Goethem C.; Stassen I.; Smolders S.; Ameloot R.; Vankelecom I.; Thielemans W.; De Vos D. E. Stabilising Ni catalysts for the dehydration-decarboxylation-hydrogenation of citric acid to methylsuccinic acid. Green Chem. 2017, 19, 4642–4650. 10.1039/C7GC01773A.

[ref32] Holzhäuser F. J.; Artz J.; Palkovits S.; Kreyenschulte D.; Büchs J.; Palkovits R. Electrocatalytic upgrading of itaconic acid to methylsuccinic acid using fermentation broth as a substrate solution. Green Chem. 2017, 19, 2390–2397. 10.1039/C6GC03153F.

[ref33] Buback M.; Müller E. Propagation Kinetics of Binary Acrylate-Methacrylate Free-Radical Bulk Copolymerizations. Macromol. Chem. Phys. 2007, 208, 581–593. 10.1002/macp.200600547.

[ref34] Drache M.; Stehle M.; Mätzig J.; Brandl K.; Jungbluth M.; Namyslo J. C.; Schmidt A.; Beuermann S. Identification of β scission products from free radical polymerizations of butyl acrylate at high temperature. Polym. Chem. 2019, 10, 1956–1967. 10.1039/C9PY00103D.

[ref35] Beuermann S.; Buback M.; Gadermann M.; Gunzler F. Depropagation in Methacrylate Polymerizations. DECHEMA Monogr. 2004, 138, 461–465.

[ref36] https://www.originlab.com/(accessed August 22, 2024).

[ref37] Brandl F.; Drache M.; Beuermann S. Kinetic Monte Carlo simulation based detailed understanding of the transfer processes in semi-batch iodine transfer emulsion polymerizations of vinylidene fluoride. Polymers 2018, 10, 1008 10.3390/polym10091008. 30960933 PMC6403726

[ref38] Schwaderer J.; Drache M.; Beuermann S. Temperature dependence of the number of defect-structures in poly(vinylidene fluoride). Molecules 2024, 29, 1551 10.3390/molecules29071551. 38611830 PMC11013231

[ref39] Metropolis N.; Rosenbluth A. W.; Rosenbluth M. N.; Teller A. H.; Teller E. Equation-of-state calculations by fast computing machines. J. Chem. Phys. 1953, 21, 1087–1092. 10.1063/1.1699114. 41342507

